# Glypican-4 trajectory predicts the risk of gestational diabetes mellitus and the requirement for insulin therapy during pregnancy

**DOI:** 10.3389/fendo.2025.1483567

**Published:** 2025-03-21

**Authors:** Lei Tang, Ping Li, Ling Li

**Affiliations:** ^1^ Department of Endocrinology, Shengjing Hospital of China Medical University, Shenyang, Liaoning, China; ^2^ Liaoning Province Key Laboratory of Endocrine Diseases, Shenyang, Liaoning, China

**Keywords:** glypican-4, gestational diabetes mellitus, oral glucose tolerance test, insulin therapy, postpartum glucose metabolism

## Abstract

**Background:**

Gestational diabetes mellitus (GDM), characterized by the onset of glucose intolerance during pregnancy, results in a series of complications for maternal and fetal health. Oral glucose tolerance test (OGTT) for screening glucose metabolism is performed in mid-to-late pregnancy, which remains less time to optimize glycemic control. Glypican-4, an insulin-sensitive adipose hormone, exhibits correlations with metabolic indicators. This study aims to investigate the association between glypican-4 and the risk of developing GDM, as well as the effects on insulin therapy and postpartum glucose metabolism.

**Methods:**

Based on pregnancy 75-g OGTT results, 718 subjects were grouped into normal glucose tolerance (NGT, n = 345) and GDM (n = 373) groups. 373 GDM patients were divided into the diet (n = 237) and insulin (n = 136) groups according to the treatment of hyperglycemia in pregnancy. Based on postpartum 75-g OGTT results, 158 of the 373 GDM patients were further divided into the NGT after delivery (NGTd, n = 138) and abnormal glucose tolerance (AGT, n = 20) groups.

**Results:**

Glypican-4 level was significantly higher in GDM than NGT subjects during pregnancy (*P*< 0.001). Glypican-4 was an independent predictor of GDM with the cut-offs were 0.40 ng/mL (5-12 weeks of gestation) and 0.79 ng/mL (13-23 weeks of gestation). Furthermore, glypican-4 level in the insulin group was higher than the diet group, which was a potential predictor of insulin therapy.

**Conclusions:**

Glypican-4 during pregnancy is associated with GDM risk, with higher levels indicating increased risk. Glypican-4 was also related to insulin therapy in GDM.

## 1 Introduction

Gestational diabetes mellitus (GDM), which constitutes an estimated 86.4% of hyperglycemic pregnancies, is characterized by the onset of glucose intolerance with identified or diagnosed for the first time during pregnancy (1, 2). As the predominant form of hyperglycemia complicating pregnancies, GDM potentially results in a series of short-term and long-term complications for both maternal and fetal health (3–5). Consequently, clinical practice guidelines underscore the necessity for stringent glycemic control as a paramount objective throughout the whole pregnancy (6, 7). However, an oral glucose tolerance test (OGTT) for screening glucose metabolism during pregnancy is administered in middle and late pregnancy, conventionally between 24 to 28 weeks of gestation, or occasionally delayed until 32 weeks. It remains less time for pregnant women at risk of GDM to optimize glycemic control through lifestyle management or medication in order to reduce the duration of elevated blood glucose exposure. Therefore, early identification and diagnosis base on some simple serological indicators, and timely and effective intervention for GDM to achieve glycemic benchmarks and create a conducive intrauterine environment is of great significance to the mother and fetus.

Glypican-4, a novel adipokine sensitive to insulin and produced by adipose tissue, plays a pivotal role in insulin signaling by binding directly to the insulin receptor (8). Gesta and colleagues firstly discovered that the expression of glypican-4 varied between the visceral and subcutaneous adipose tissues of rodents and humans (9). Intriguingly, this expression pattern significantly correlated with several measures of obesity, including body fat content, body mass index (BMI) and waist-to-hip ratio. Additionally, it showed an independent association with insulin resistance (IR), as measured by euglycemic hyperinsulinemic clamps. Of greater significance, serum levels of glypican-4 have been found to be linked to key metabolic indicators such as the ratio of visceral to subcutaneous fat area, fasting plasma insulin (FINS) and the homeostasis model assessment of insulin resistance (HOMA-IR), highlighting its potential as a biomarker for metabolic disorders associated with obesity and IR (10, 11).

However, no longitudinal studies have documented the serum glypican-4 trajectories in GDM patients, particularly in the context of insulin therapy or postpartum glucose metabolism. In the present study, we address this gap by examining a cohort of pregnant women from Northeast China, to elucidate the association between glypican-4 and the risk of developing GDM, as well as its potential effects on insulin therapy and postpartum glucose metabolism in GDM patients for the first time.

## 2 Materials and methods

### 2.1 Study participants

Pregnant women who underwent regular prenatal care and postpartum follow-up at the Endocrinology Outpatient Clinic of Shengjing Hospital of China Medical University between December 2017 and August 2023, were recruited. None of the participants had previous diabetes, any other diseases affecting blood glucose levels, such as hyperthyroidism, Cushing syndrome, pancreatitis and pancreatic exocrine disease, taken glucocorticoids and other glucose-altering medications, other comorbidities such as asthma, malignancies, autoimmune diseases, and severe cardiorenal disorders. Enrolled participants had provided general characteristics, and completed physical examinations and blood measurements during pregnancy and after delivery. Finally, a total of 718 singleton pregnant women with complete data and complying with the criteria were enrolled for further analysis. The study protocol was approved by the ethics committee of Shengjing Hospital of China Medical University (2023PS38K).

718 enrolled participants were categorized into two groups according to the 75-g OGTT results during pregnancy: GDM (n = 373) and normal glucose tolerance (NGT, n = 345). GDM was defined if participants with one or more of the pathological glucose values in 75-g OGTT: 5.1 mmol/L ≤ fasting plasma glucose (FPG)< 7.0 mmol/L, 1-h plasma glucose ≥ 10.0 mmol/L, and 8.5 mmol/L ≤ 2-h plasma glucose< 11.1 mmol/L (12, 13). Furthermore, 373 patients with GDM were grouped into the diet group (n = 237) and insulin group (n = 136) based on the treatment of hyperglycemia in pregnancy.

The 75-g OGTT results screened for glucose metabolism after delivery in our hospital was utilized to further stratify 158 of the 373 patients with GDM into the abnormal glucose tolerance (AGT, n = 20) and NGT after delivery (NGTd, n = 138) groups. Postpartum glucose metabolism was evaluated based on World Health Organization criteria (14), with type 2 diabetes mellitus (T2DM) diagnosed when participants with typical diabetes symptoms and either of the abnormal glucose values. Participants without typical diabetes symptoms were also diagnosed with T2DM using two confirmed blood glucose. Impaired fasting glucose (IFG) and impaired glucose tolerance (IGT) were defined according to specific FPG and 2-h plasma glucose thresholds, with individuals exhibiting either condition classified as pre-diabetes.

### 2.2 Clinical data

Clinical and biochemical data pertaining to the study participants were meticulously archived within the Hospital Information System (version 5.0). A comprehensive maternal profile was obtained by trained medical professionals, encompassing demographic and obstetric history, such as last menstrual period, maternal age, educational status, a family history of diabetes, menstrual history, parity, prior GDM, history of macrosomia in previous pregnancies, details of previous adverse pregnancy outcomes, polycystic ovary syndrome (PCOS), height, pre-gestational weight and BMI, and acanthosis nigricans. BMI was calculated as weight (kg)/height^2^ (m^2^). Management of hyperglycemia, insulin dosage and obstetric complications were also documented throughout the pregnancy. Prenatal weight, delivery time and methods, neonatal weight, gender, Apgar scores and feeding modalities, and adverse neonatal outcomes such as mortality, malformations, hypoglycemia, pathologic jaundice, and respiratory distress were obtained after delivery.

The 75-g OGTT was conducted in accordance with the World Health Organization criteria. Collected fasting blood samples were also used to detect other metabolic indicators, including serum triglycerides (TG), high-density lipoprotein cholesterol, low-density lipoprotein cholesterol, alanine aminotransferase, aspartate aminotransferase, free triiodothyronine (FT3), free thyroxine (FT4), thyroid-stimulating hormone (TSH), thyroglobulin antibodies (TgAb) and thyroid peroxidase antibodies (TPOAb).

For patients with GDM, FPG, 2-h postprandial plasma blood glucose after breakfast and lunch, and glycated albumin (GA) were monitored every 2-3 weeks; hemoglobin A1c (HbA1c) was evaluated every 2-3 months; and self-monitoring of blood glucose was employed to appraise the efficacy of therapeutic interventions. Lifestyle intervention was the cornerstone of treatment for gestational hyperglycemia, with personalized nutritional plans designed to ensure adequate caloric intake for optimal fetal and maternal health, prioritizing foods with a low glycemic index. Physical activity was prescribed, consisting of aerobic and/or resistance exercises not exceeding 45 minutes daily.

Insulin therapy was initiated along with a continuation of lifestyle management in the event of uncontrolled glucose. The targeted glucose control parameters during pregnancy were fingerprick fasting blood glucose 3.3-5.3 mmol/L, 1-h postprandial blood glucose less than 7.8 mmol/L, and 2-h postprandial blood glucose less than 6.7 mmol/L.

Calculation formulas for glucose metabolism indicators, including HOMA-IR, the homeostatic model assessment indices for β-cell function (HOMA-β), as well as the composite insulin sensitivity index (CISI), were calculated as follows (15, 16):

$\text{HOMA-IR}=\frac{\text{FINS (μU/mL)}\times \text{FPG (mmol/L)}}{22.5}$

$\text{HOMA-β}=\frac{20\times \text{FINS (μU/mL)}}{\text{ FPG (mmol/L) }-3.5}$


$$\begin{array}{c} \text{ CISI}=\\ \frac{10000}{\sqrt{\text{ [FPG (mmol/L) }\times \text{FINS (μU/mL)}]\;\times \;[{\text{meanGLU}}_{\text{OGTT}}\;\left(\text{mmol}/\text{L}\right)\;\times {\text{meanINS}}_{\text{OGTT}}\text{ (μU/mL)}]}} \end{array}$$

### 2.3 Glypican-4 measurement

Serum levels of glypican-4 were quantified employing a commercially available enzyme-linked immunosorbent assay kit (CCC, USA). The assay’s precision was ascertained through the intra-assay and inter-assay coefficients of variation, which were established to be less than 10% and 12%, respectively. Additionally, the enzyme activity reduction rate was determined to be less than 5%, thereby attesting to the robustness and reproducibility of this procedure.

### 2.4 Statistical analysis

Data management and statistical analyses were conducted utilizing SPSS software version 22.0 (IBM Corporation, Armonk, NY, USA) and GraphPad Prism version 6 (GraphPad Software, Inc., San Diego, CA, USA). Variables adhering to a normal distribution were expressed as mean ± standard deviation, whereas those exhibiting a skewed distribution were shown as median (interquartile range). For the comparison of continuous variables between groups, the Student’s t-test was applied for normally distributed data, and the Mann-Whitney U test was utilized for data of skewed distribution. Categorical data were presented as percentages and subjected to the Chi-square test for group comparisons. Correlations between glypican-4 and metabolic variables were evaluated. Logistic regression models were developed to assess the association of glypican-4 with GDM and insulin therapy during pregnancy. The Receiver Operating Characteristic (ROC) curve analysis was employed to ascertain the optimal cut-off points for diagnosing GDM and predicting the requirement of insulin therapy during pregnancy. Statistical significance was defined by a *P*-value of less than 0.05.

## 3 Results

### 3.1 Association between glypican-4 during pregnancy and GDM

#### 3.1.1 Characteristics and clinical biochemical indicators

Of the 718 participants, 345 had normal glucose tolerance test results and 373 were diagnosed with GDM. Maternal age, pre-gestational weight and maximum weight, pre-gestational BMI, a family history of diabetes, acanthosis nigricans, previous adverse pregnancy outcomes, and prenatal weight in the GDM group were higher than in the NGT group, while weight gain per week in the GDM group was lower compared with the NGT group (*P*< 0.05, **Table 1**).

**Table 1:** Characteristics of participants.

	NGT (n=345)	GDM (n=373)	*P*
Maternal age (years)	30.5 ± 3.8	31.2 ± 4.2 ^a^	0.015
Pre-gestational weight (kg)	59.4 ± 10.8	65.7 ± 12.5 ^b^	<0.001
Pre-gestational BMI (kg/m^2^))	22.8 ± 4.1	25.0 ± 4.4 ^b^	<0.001
Pre-gestational maximum weight (kg)	61.3 ± 11.7	67.5 ± 13.4 ^b^	<0.001
A family history of diabetes (n/%)	66 (19.1%)	150 (40.2%) ^b^	0.006
History of polycystic ovary syndrome (n/%)	28 (8.1%)	37 (9.9%)	0.637
Acanthosis nigricans (n/%)	23 (6.7%)	102 (27.3%) ^b^	0.001
Previous adverse pregnancy outcomes (n/%)	19 (5.5%)	102 (27.3%) ^b^	<0.001
Prenatal weight (kg)	73.8 ± 11.8	75.9 ± 11.5 ^a^	0.019
Weight gain per week (kg)	0.4 ± 0.2	0.3 ± 0.2 ^b^	0.001
Delivery time (weeks)	38.6 ± 1.3	38.4 ± 1.1	0.553
Birth weight of newborns (g)	3517 ± 345	3386 ± 519	0.477

Data are presented as mean ± standard deviation or percentages. NGT, normal glucose tolerance; GDM, gestational diabetes mellitus; BMI, body mass index. ^a^ *P<*0.05, ^b^ *P<*0.01 vs the NGT group.

A comparison of glucose metabolism indicators during pregnancy between the NGT and GDM groups was presented in **Table 2**. Among the biochemical parameters measured at the first prenatal visit, serum FT3 (4.51 [4.20, 4.78] vs 4.29 [3.97, 4.72] pmol/L, *P*< 0.001), TgAb (3.27 [1.02, 42.56] vs 0.17 [0.00, 1.05] IU/mL, *P*< 0.001), and TPOAb (2.02 [1.05, 32.75] vs 0.60 [0.18, 2.04] IU/mL, *P*< 0.001) levels in the GDM group were higher compared with the NGT group; TG (2.15 [1.37, 3.73] vs 1.78 [0.97, 2.60] mmol/L, *P* = 0.025) level was also higher in the GDM group. However, no other statistically significant differences in metabolic indicators were observed between the two groups (**Table 2**).

**Table 2:** Comparison of clinical biochemical indicators.

	NGT (n=345)	GDM (n=373)	*P*
Glucose metabolism indicators at GDM diagnosis			
Gestational period (weeks)	20.1 ± 7.8	19.8 ± 8.1	0.332
Fasting plasma glucose (mmol/L)	4.6 (4.5, 4.9)	5.5 (5.2, 6.1) ^b^	<0.001
1-h plasma glucose (mmol/L)	7.6 (6.9, 8.7)	10.4 (9.0, 11.7) ^b^	<0.001
2-h plasma glucose (mmol/L)	6.5 (5.6, 7.4)	8.7 (7.1, 10.2) ^b^	<0.001
Fasting plasma insulin (μU/mL)	7.5 (5.8, 9.0)	11.8 (9.3, 15.8) ^b^	<0.001
1-h plasma insulin (μU/mL)	50.2 (34.0, 75.1)	63.9 (44.7, 107.7)	0.054
2-h plasma insulin (μU/mL)	44.1 (28.2, 60.3)	67.4 (43.3, 113.9) ^b^	0.003
GA (%)	11.7 (10.6, 13.0)	13.0 (11.8, 14.3) ^b^	<0.001
HbA1c (%)	4.9 (4.7, 5.2)	5.4 (5.1, 7.8) ^b^	<0.001
HOMA-IR	1.5 (1.2, 1.9)	2.8 (2.3, 4.0) ^b^	<0.001
HOMA-β	146.9 (105.9, 175.9)	122.4 (86.9, 168.1)	0.265
CISI	126.8 (88.7, 153.6)	63.2 (38.0, 88.5) ^b^	<0.001
Other biochemical indicators			
FT3 (pmol/L)	4.29 (3.97, 4.72)	4.51 (4.20, 4.78) ^b^	<0.001
TSH (uIU/mL)	1.7132 (0.9224, 2.8479)	2.0300 (0.7036, 3.3038)	0.294
TPOAb (IU/mL)	0.60 (0.18, 2.04)	2.02 (1.05, 32.75) ^b^	<0.001
TgAb (IU/mL)	0.17 (0.00, 1.05)	3.27 (1.02, 42.56) ^b^	<0.001
TG (mmol/L)	1.47 (1.02, 2.64)	2.09 (1.44, 2.94) ^a^	0.030
LDL-C (mmol/L)	2.53 (2.02, 3.26)	2.76 (2.33, 3.15)	0.621

Data are presented as mean ± standard deviation or median (interquartile range). NGT, normal glucose tolerance; GDM, gestational diabetes mellitus; GA, glycated albumin; HbA1c, hemoglobin A1c; HOMA-IR, homeostasis model assessment of insulin resistance; HOMA-β, homeostasis model assessment of β cells; CISI, composite insulin sensitivity index; FT3, free triiodothyronine; TSH, thyroid stimulating hormone; TPOAb, thyroid peroxidase antibody; TgAb, thyroglobulin antibody; TG, triglyceride; LDL-C, low density lipid-cholesterol. ^a^ *P<*0.05, ^b^ *P<*0.01 vs the NGT group.

#### 3.1.2 Longitudinal changes and comparison of glypican-4 during pregnancy

Serum glypican-4 levels during pregnancy were measured in four phases, corresponding to the following gestational weeks: 8.6 ± 2.3, 18.2 ± 3.3, 26.2 ± 1.5 and 31.3 ± 2.2 weeks.

**Figure 1:**
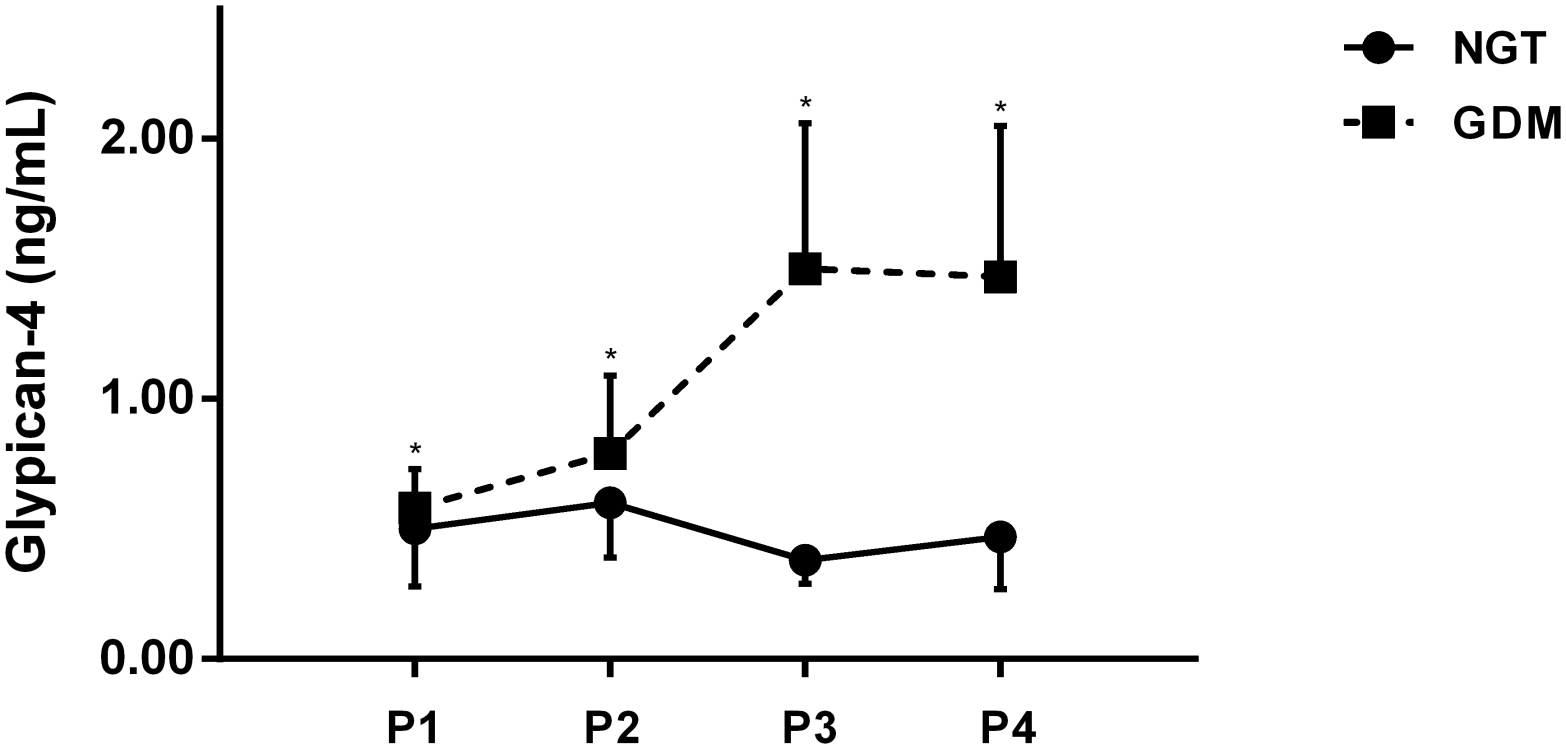
Longitudinal changes and comparison among glypican-4 during pregnancy NGT, normal glucose tolerance; GDM, gestational diabetes mellitus; P1, 5-12 weeks of gestation; P2, 13-23 weeks of gestation; P3, 24-28 weeks of gestation; P4, 29-40 weeks of gestation. * P<0.05 vs the NGT group.

As shown in **Figure 1**, serum glypican-4 levels were higher in the GDM group compared to the NGT group throughout the whole pregnancy, with significant differences observed during 24-28 weeks of gestation and 29-40 weeks of gestation (*P*< 0.05).

#### 3.1.3 Correlation between glypican-4 and glucose metabolism indicators

Glypican-4 level during pregnancy was positively correlated with FPG, 1-h and 2-h plasma glucose, GA and HbA1c at the time of GDM diagnosis. No significant correlation was observed with other indicators (*P* > 0.05, **Table 3**).

**Table 3:** Correlation between glypican-4 and glucose metabolism indicators.

	Glypican-4	
	*r*	*P*
Fasting plasma glucose	0.367 ^a^	<0.001
1-h plasma glucose	0.299 ^a^	<0.001
2-h plasma glucose	0.334 ^a^	<0.001
Fasting plasma insulin	0.124	0.189
1-h plasma insulin	0.099	0.327
2-h plasma insulin	0.179	0.073
GA	0.132 ^a^	<0.001
HbA1c	0.186 ^a^	0.001
HOMA-IR	0.150	0.112
HOMA-β	-0.021	0.825
CISI	-0.113	0.317

GA, glycated albumin; HbA1c, hemoglobin A1c; HOMA-IR, homeostasis model assessment of insulin resistance; HOMA-β, homeostasis model assessment of β cells; CISI, composite insulin sensitivity index. *r*: correlation. ^a^ *P<*0.05.

#### 3.1.4 Risk analysis of GDM

In the logistic regression model, maternal age (odds ratio [OR] = 1.048, 95% confidence interval [CI]: 1.009-1.089, *P* = 0.015), pre-gestational BMI (OR = 1.136, 95%CI: 1.088-1.186, *P*< 0.001), a family history of diabetes (OR = 1.959, 95%CI: 1.343-2.858, *P*< 0.001), acanthosis nigricans (OR = 3.490, 95%CI: 2.183-5.578, *P*< 0.001), previous adverse pregnancy outcomes (OR = 1.042, 95%CI: 1.026-1.058, *P*< 0.001) and prenatal weight (OR = 1.048, 95%CI: 1.032-1.064, *P<* 0.001) were potential predictors of GDM. Adjusted for confounding factors using a stepwise logistic regression, glypican-4 was identified as an independent predictor of GDM, with an increasing level correlating to a heightened risk of GDM (**Table 4**).

**Table 4:** Association of glypican-4 during pregnancy with GDM.

	Glypican-4 in 5-12 weeks of gestation			
	1st	2nd	3rd	4th
glypican-4	0.23 (0.14, 0.30)	0.46 (0.40, 0.50)	0.62 (0.58, 0.66)	0.82 (0.76, 0.93)
Model 1	1.0 (reference)	1.385 (1.013, 1.919) ^a^	3.909 (1.204, 8.609) ^a^	5.104 (2.826, 31.552) ^b^
Model 2	1.0 (reference)	1.138 (1.026, 1.263) ^a^	1.461 (1.031, 1.742) ^a^	4.783 (1.071, 21.359) ^a^
Model 3	1.0 (reference)	1.059 (0.965, 1.162)	1.341 (0.965, 1.864)	4.456 (1.053, 18.849) ^a^
Model 4	1.0 (reference)	1.032 (0.995, 1.070)	1.314 (0.935, 1.848)	4.400 (1.022, 18.949) ^a^

	Glypican-4 in 13-23 weeks of gestation			
	1st	2nd	3rd	4th
glypican-4	0.28 (0.16, 0.36)	0.52 (0.44, 0.59)	0.79 (0.71, 0.84)	1.34 (1.08, 1.69)
Model 1	1.0 (reference)	1.379 (1.047, 1.818) ^a^	2.899 (1.460, 5.755) ^b^	3.183 (1.124, 9.012) ^b^
Model 2	1.0 (reference)	1.360 (1.023, 1.807) ^a^	1.148 (1.054, 1.252) ^b^	2.815 (1.372, 5.776) ^b^
Model 3	1.0 (reference)	1.056 (1.022, 1.092) ^b^	1.138 (1.043, 1.240) ^b^	2.553 (1.157, 5.634) ^a^
Model 4	1.0 (reference)	1.041 (0.964, 1.124)	1.061 (1.026, 1.096) ^b^	2.453 (1.102, 5.458) ^a^

GDM, gestational diabetes mellitus. Model: logistic regression analysis. Model 1: no adjustment; Model 2: Model 1+adjusting for maternal age, and pre-gestational BMI; Model 3: Model 2+adjusting for family history of diabetes, acanthosis nigricans, and previous adverse pregnancy outcomes; Model 4: Model 3+adjusting for prenatal weight. ^a^ P <0.05, ^b^ P <0.01 vs the ﬁrst interquartile.

**Figure 2:**
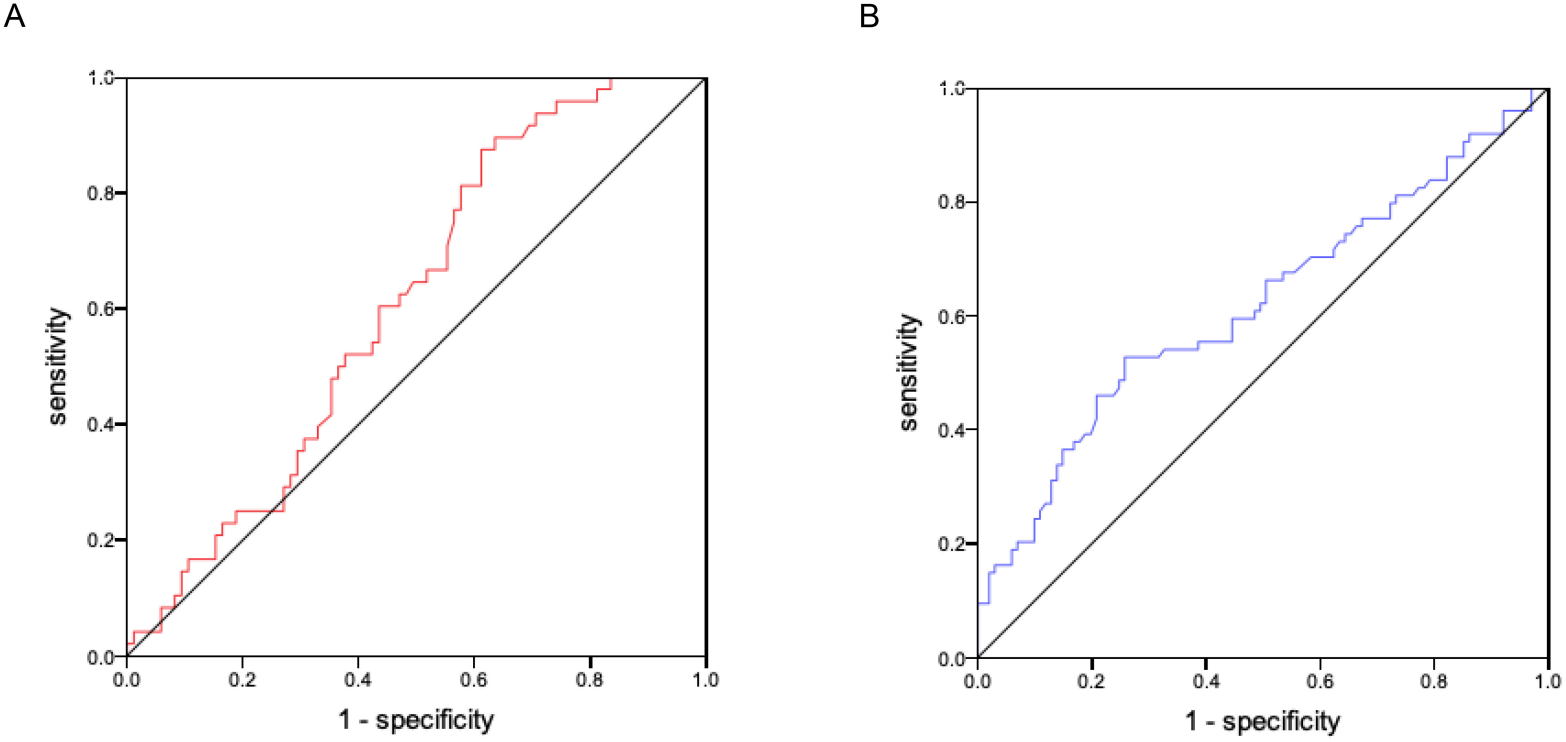
Receiver operating characteristics curve of glypican-4 for gestational diabetes mellitus **(A)**. Receiver operating characteristics curve of glypican-4 in 5-12 weeks of gestation. **(B)** Receiver operating characteristics curve of glypican-4 in 13-23 weeks of gestation. The red and blue lines represent glypican-4 in 5-12 and 13-23 weeks of gestation, respectively. The black line is the reference line.

ROC curves were used to establish the cut-off value of glypican-4 to predict GDM (**Figure 2**). As shown in **Figure 2A**, the cut-off value of glypican-4 in 5-12 weeks of gestation was 0.40 ng/mL (sensitivity 87.5% and specificity 38.8%), with an area under the curve (AUC) of 0.609 (95%CI: 0.514-0.707, *P* = 0.037). As shown in **Figure 2B**, the cut-off value of glypican-4 in 13-23 weeks of gestation was 0.79 ng/mL (sensitivity 52.7% and specificity 74.3%), with an AUC of 0.618 (95%CI: 0.531-0.704, *P* = 0.008).

### 3.2 Association between glypican-4 during pregnancy and insulin therapy in patients with GDM

#### 3.2.1 Comparison of glypican-4 during pregnancy

Among the 373 patients with GDM, 237 of them achieved glycemic control through lifestyle intervention alone, while an additional 136 required insulin therapy in conjunction with medical nutrition therapy. Serum glypican-4 (1.41 [0.73, 1.99] vs. 0.85 [0.54, 1.77] ng/mL, *P* = 0.001) levels in the insulin group were higher than in the diet group.

#### 3.2.2 Risk analysis of insulin therapy during pregnancy

Stepwise logistic regression analysis adjusting for confounders indicated that glypican-4 was a potential predictor of insulin therapy during pregnancy, with a significant increase in insulin requirement as glypican-4 levels increased (**Table 5**).

**Table 5:** Association of glypican-4 during pregnancy with insulin therapy.

	Glypican-4 in 5-23 weeks' gestation			
	1st	1st	1st	1st
glypican-4	0.43 (0.32, 0.52)	0.79 (0.66, 0.86)	1.50 (1.34, 1.63)	2.15 (2.05, 2.62)
Model 1	1.0 (reference)	1.423 (1.123, 1.801) ^b^	1.872 (1.283, 2.732) ^b^	2.838 (1.682, 4.787) ^b^
Model 2	1.0 (reference)	1.360 (1.053, 1.758) ^b^	1.721 (1.133, 2.616) ^b^	2.255 (1.284, 3.960) ^b^
Model 3	1.0 (reference)	1.358 (1.070, 1.723) ^b^	1.700 (1.160, 2.491) ^b^	1.935 (1.122, 3.337) ^b^
Model 4	1.0 (reference)	1.079 (1.024, 1.136) ^b^	1.419 (1.162, 1.733) ^b^	1.845 (1.344, 2.532) ^b^

Model: logistic regression analysis. Model 1: no adjustment; Model 2: Model 1+adjusting for maternal age, and pre-gestational BMI; Model 3: Model 2+adjusting for family history of diabetes, acanthosis nigricans, and previous adverse pregnancy outcomes; Model 4: Model 3+adjusting for prenatal weight. ^a^ P <0.05, ^b^ P <0.01 vs the ﬁrst interquartile.

**Figure 3:**
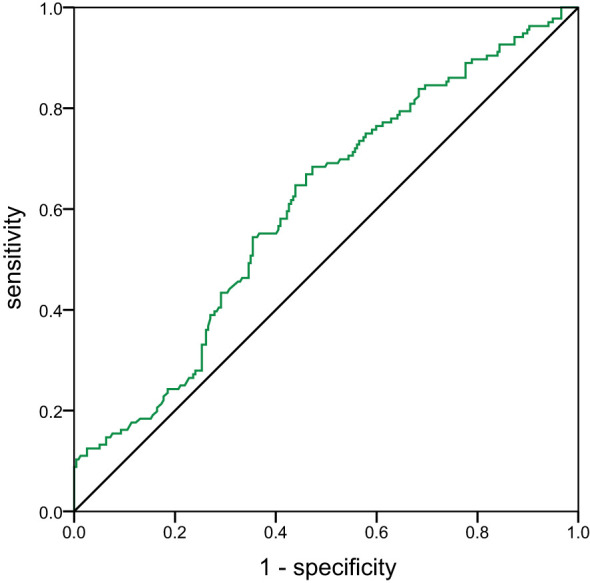
Receiver operating characteristics curve of glypican-4 for insulin therapy during pregnancy The green line represents glypican-4 during pregnancy. The black line is the reference line.

ROC curves were conducted to determine the cut-off value of glypican-4 for insulin therapy during pregnancy (**Figure 3**). The cut-off value of glypican-4 was 0.87 ng/mL (sensitivity 68.4% and specificity 52.7%), with an AUC of 0.606 (95% CI: 0.547-0.665, *P* = 0.001).

### 3.3 Analysis of glypican-4 during pregnancy affecting postpartum blood glucose

Of 158 GDM participants who underwent a postpartum 75-g OGTT after delivery in our hospital, 138 exhibited normal results, whereas 20 (12.7%) displayed abnormal glucose tolerance test outcomes (18 had pre-diabetes and 2 had T2DM). The mean interval from delivery to postpartum OGTT was 12.0 ± 7.5 weeks, at a screening rate of 42.4%.

Serum glypican-4 (0.85 [0.50, 1.15] vs. 0.65 [0.39, 0.95] ng/mL, *P* = 0.316) levels in the AGT group were higher than in the NGTd group throughout the whole pregnancy; however, the difference was not statistically significant.

## 4 Discussion

The latest IDF data have revealed that the prevalence of GDM in pregnancies worldwide ranges from 7.1% to 27.6% (with a global prevalence of 14.0%), attributed to the increasing maternal age and obesity rates (1). Therefore, early identification of hyperglycemia in pregnancy is crucial for preventing adverse events. Currently, the precise etiological mechanism underlying GDM remains unclear; however, a large number of studies have indicated that IR is considered the hallmark of GDM pathogenesis (17, 18). It is widely acknowledged that insulin, a key hypoglycemic hormone, must bind with the insulin receptor to exert its biological effects through a series of intricate enzymatic reactions. Any abnormality in the involved signal cascade reaction could lead to IR (19, 20). IR can reduce the responsiveness of target organs or peripheral tissues to insulin, thereby affecting glucose output, uptake and utilization, as well as fat and protein synthesis and energy metabolism (20, 21). Under normal pregnancy, insulin sensitivity in target tissues such as fat tissue and skeletal muscle, and glucose handling capacity by insulin are lower compared with the pre-pregnancy status; however, the compensatory increase in insulin secretion could maintain normal blood glucose levels. By contrast, the degree of IR in individuals with GDM is significantly higher than that of normal healthy pregnant women, and the pancreatic β-cell dysfunction and the progressive decrease in insulin secretion cannot compensate for the increase in IR (17, 18).

Glypicans, a class of heparan sulfate proteoglycans, are anchored to the extracellular surface of the plasma membrane via glycosyl-phosphatidylinositol (GPI) linkages and have played a pivotal role in modulating signaling pathways, including those of Wnt, Hedgehog, fibroblast growth factors, and bone morphogenetic proteins (22). Glypican-4, a member of this GPI-anchored proteoglycan family, is synthesized and secreted by adipose tissue and has been shown to interact directly with the insulin receptor, modulating its activation and the subsequent signaling cascade (22). The regulatory mechanism involving glypican-4 and insulin sensitivity is linked to GPI-specific phospholipase D (GPLD), an enzyme implicated in the release of glypican-4 from the cell surface into circulation, with its activity being subject to regulation by insulin (23). Increased insulin levels have been demonstrated to upregulate GPLD-1 activity, leading to enhanced glypican-4 shedding from the cell surface and consequently, elevated levels of circulating glypican-4, which has been correlated with IR (22).

Gesta et al. reported elevated serum glypican-4 levels in insulin-resistant obese subjects, with a twofold increase compared to those with insulin sensitivity, matched for age, sex, and BMI, and noted a positive association with both FINS and HOMA-IR (9). Zhu et al. also observed that obese individuals with IR have higher serum glypican-4 levels, and glypican-4 was closely related to a variety of metabolic-related parameters, including BMI, systolic blood pressure, alanine aminotransferase, aspartate aminotransferase, FINS and HOMA-IR (10). A recent adult study demonstrated that individuals with metabolic syndrome (MetS) exhibited significantly elevated serum glypican-4 levels when contrasted with those of healthy and non-MetS counterparts (11). An elevated glypican-4 level could be an independent risk for MetS in the Chinese Han population, indicating a heightened likelihood of MetS development among individuals with increased levels (11). In concordance with prior research, serum glypican-4 levels were significantly elevated in individuals with impaired glucose tolerance (24). Conversely, serum glypican-4 levels were paradoxically lower in newly diagnosed T2DM patients compared to those with normal pregnant women, and reduced glypican-4 levels also observed in an ob/ob mice model with elevated blood glucose and hyperinsulinemia, suggesting a complex role for glypican-4 in glucose metabolism dysregulation (8, 24). Yoo et al. further delineated sex-specific disparities in serum glypican-4 levels within Asian cohorts and a significant correlation with cardiometabolic risk factors, such as body fat distribution, arterial stiffness, and IR (25). Elevated serum glypican-4 levels have also been observed in women with nonalcoholic fatty liver disease, indicative of its hepatic involvement in MetS (25). Additionally, in women with PCOS presenting metabolic risk factors, higher glypican-4 levels were associated with established cardiovascular disease predictors, including IR, hyperglycemia, hypertension, and dyslipidemia, with a notable emphasis on adipose tissue distribution (26). Serum glypican-4 levels have also been reported to escalate with the severity of obesity in pediatric populations aged 6 to 18 years (27). Collectively, these findings underscore the important role of glypican-4 in obesity and IR, and its utility as a metabolic disorder biomarker. However, no existing evidence has reported the longitudinal data on glypican-4 trajectories in GDM, particularly in the context of insulin therapy or postpartum. This study pioneers the examination of the association between maternal glypican-4 levels and GDM risk, as well as its predictive value for insulin therapy requirements and postpartum glycemic outcomes.

Our study showed that serum glypican-4 levels in GDM patients were higher than healthy pregnant women in different trimesters throughout the whole pregnancy, with significant differences observed in middle and late pregnancy. The study by Deischinger et al. also reported that glypican-4 levels in GDM patients during pregnancy increased progressively with advancing gestational weeks, but no differences were noted in comparison to healthy pregnant women (28). The study was limited by a smaller number of participants, all of whom were obese with higher BMI, differing significantly from the demographic in our study, which may account for the lack of observed differences between the groups. It is widely recognized that maternal IR progressively deteriorates throughout pregnancy, becoming more pronounced during the mid-to-late pregnancy and reaching a peak at approximately 32 weeks of gestation. Compared to healthy pregnant women, patients with GDM experience more severe IR. Our study found that serum glypican-4 levels were significantly elevated in GDM patients during the second and third trimesters of pregnancy, which was consistent with the characteristics of changes in IR during pregnancy, and exhibited the potential of glypican-4 as a serological marker associated with IR. Moreover, maternal glypican-4 levels during pregnancy demonstrated a significant positive correlation with key diagnostic indicators of glucose metabolism at the time of GDM diagnosis in our study. An elevation in glypican-4 levels was associated with an increased risk of GDM, with a statistically higher risk observed in pregnant women with glypican-4 levels exceeding 0.40 ng/mL in the first trimester (5-12 weeks of gestation) or 0.79 ng/mL in the second trimester (13-23 weeks of gestation). These results suggest that glypican-4 during pregnancy was closely associated with glucose metabolism and highlighted the potential of glypican-4 as a predictive serum marker for the identification and clinical diagnosis of GDM.

Medical nutrition therapy is a fundamental intervention for managing hyperglycemia in pregnancy, which followed individualization principle (6, 13). The intervention principles should ensure appropriate nutrition and calories intake of pregnant women, normal growth and development of the fetus, and better blood glucose maintenance to avoid ketosis (6, 13). The majority of patients with GDM can achieve optimal glycemic control through evidence-based dietary and exercise interventions; however, for those who fail to effectively control blood glucose, healthcare providers encounter a significant challenge in the early identification of high-risk cohorts and the strategic initiation of insulin therapy.

In our current study, 136 of the 373 patients with GDM necessitated supplementary insulin therapy in conjunction with ongoing lifestyle interventions to attain the predefined glycemic control targets. We found that serum glypican-4 levels in GDM with requirement for insulin therapy were higher than them with medical nutrition therapy only. Glypican-4 was a potential predictor of insulin therapy during pregnancy, a significantly increased insulin requirement with increased glypican-4 levels. It was more necessary for GDM with glypican-4 exceeding 0.87 ng/mL to require insulin therapy during pregnancy to manage blood glucose.

To the best of our knowledge, women with a history of GDM are considered to be at a significantly elevated risk for the development of diabetes, with a nearly 10 times than healthy women (29, 30). However, currently only a few patients with GDM are screened for diabetes after delivery. It is largely attributed to the insufficient understanding of diabetes and the perceived complexity of OGTT, leading to a reduced rate for early detection of postpartum glucose disorders and a subsequent increased incidence of long-term diabetes. A large retrospective study had demonstrated a significant escalation in the cumulative incidence of diabetes, rising from 2.6% at 6 weeks to over 70% within 28 years postpartum, with a particularly pronounced increase within the first five years postpartum, a period recognized as a critical window for the development of diabetes (31). Furthermore, GDM patients also confront an approximately fourfold higher risk of prediabetes, encompassing IFG and/or IGT, compared to the general female population (32). Therefore, our study determined the relationship between glypican-4 level during pregnancy and subsequent postpartum glucose metabolism in patients with GDM. A cohort of 158 GDM participants were consecutively enrolled to undergo a 75-g OGTT as part of their postpartum evaluation, of which 18 had pre-diabetes and 2 had T2DM. Participants with abnormal glucose tolerance test results had higher glypican-4 level than healthy controls throughout the whole pregnancy; however, the observed difference failed to reach statistical significance, potentially attributable to the smaller postpartum follow-up sample size and the inadequate follow-up time for OGTT assessment of glucose metabolism.

This study has several advantages. First, the trajectories of maternal serum glypican-4 concentrations throughout the whole pregnancy are first measured in this longitudinal study. Second, our study presents the first comprehensive examination of the association between serum glypican-4 levels and the risk of GDM development. Simultaneously, it elucidates the biomarker’s predictive utility in ascertaining the requirement for insulin therapy and profiling the postpartum glycemic status. However, this study also has certain limitations. Our investigation has a constrained sample size screening for glucose metabolism after delivery and lacks postpartum data on glypican-4 level. Consequently, it is limited in fully exploring the relationship between glypican-4 and the long-term risk of developing metabolic disorders such as diabetes.

In conclusion, our study introduces novel findings that establish a significant association between maternal glypican-4 levels during pregnancy and the risk of GDM development. We observe a substantial increase in GDM risk with elevated glypican-4 levels, particularly when glypican-4 levels exceeding 0.40 ng/mL in the first trimester (5-12 weeks of gestation) or 0.79 ng/mL in the second trimester (13-23 weeks of gestation). Importantly, a glypican-4 level above 0.87 ng/mL is identified as a critical threshold for GDM patients necessitating insulin therapy to maintain glycemic control during pregnancy. These insights hold profound clinical implications, enabling healthcare providers to swiftly and accurately identify pregnant women at high risk of GDM. Early and accurate diagnosis, coupled with timely intervention, is essential for the management of blood glucose levels and the safeguarding of both maternal and fetal health. It is imperative to enhance awareness among these women regarding the importance of regular glucose metabolism screening and adherence to lifestyle modifications throughout their lives, as preventive measures against the development of T2DM. Furthermore, as a potential serological indicator, further research into glypican-4 is conducive to comprehensively understand the pathophysiological mechanisms of GDM.

## Data Availability

The datasets presented in this article are not readily available because the datasets analyzed during the current study are available from the corresponding author upon reasonable request. Requests to access the datasets should be directed to liling8864@hotmail.com.
